# Mapping the Peds QL^TM^ 4.0 onto CHU-9D: a cross-sectional study in functional dyspepsia population from China

**DOI:** 10.3389/fpubh.2023.1166760

**Published:** 2023-05-31

**Authors:** Qiqi Wang, Chuchuan Wan, Maozhen Li, Yuankai Huang, Xiaoyu Xi

**Affiliations:** The Research Center of National Drug Policy & Ecosystem, China Pharmaceutical University, Nanjing, China

**Keywords:** mapping, CHU-9D, Peds QL 4.0, health utility value, functional dyspepsia

## Abstract

**Objective:**

The study aims to develop a mapping algorithm from the Pediatric Quality of Life Inventory™ 4. 0 (Peds QL 4.0) onto Child Health Utility 9D (CHU-9D) based on the cross-sectional data of functional dyspepsia (FD) children and adolescents in China.

**Methods:**

A sample of 2,152 patients with FD completed both the CHU-9D and Peds QL 4.0 instruments. A total of six regression models were used to develop the mapping algorithm, including ordinary least squares regression (OLS), the generalized linear regression model (GLM), MM-estimator model (MM), Tobit regression (Tobit) and Beta regression (Beta) for direct mapping, and multinomial logistic regression (MLOGIT) for response mapping. Peds QL 4.0 total score, Peds QL 4.0 dimension scores, Peds QL 4.0 item scores, gender, and age were used as independent variables according to the Spearman correlation coefficient. The ranking of indicators, including the mean absolute error (MAE), root mean squared error (RMSE), adjusted R^2^, and consistent correlation coefficient (CCC), was used to assess the predictive ability of the models.

**Results:**

The Tobit model with selected Peds QL 4.0 item scores, gender and age as the independent variable predicted the most accurate. The best-performing models for other possible combinations of variables were also shown.

**Conclusion:**

The mapping algorithm helps to transform Peds QL 4.0 data into health utility value. It is valuable for conducting health technology evaluations within clinical studies that have only collected Peds QL 4.0 data.

## Introduction

Functional dyspepsia (FD) is a clinical syndrome occurring in the gastroduodenal region. FD is a common pediatric disorder with a prevalence of ~3% in China (1) and ~3 to 7.6% in other countries (2, 3). Children and adolescents with FD are often associated with symptoms such as postprandial fullness, early satiation, anorexia, belching, nausea, vomiting, upper abdominal gaseous distension, pain, burning sensation, and acid regurgitation (1, 4).

Previous studies have shown that FD leads to lower health-related quality of life (HRQoL) in children and adolescents, with significant negative effects on their lives (3, 5), causing sleep disturbances, psychological distress, frequent absence from school, and less social interaction (6–8). Thus, accurate measuring and monitoring of the HRQoL of patients are valuable for understanding the harm caused by FD and managing the disease. Meanwhile, it promotes the development of health technology assessment. As a key indicator for measuring HRQoL, health utility value (HUV) can adequately reflect individual preferences and has been widely used. HUVs can be obtained by direct or indirect measurement. Indirect measures are more commonly used because of their simplicity and ease of use. Currently, the main scales that indirectly measure the quality of life and HUVs in children or adolescents include the Child Health Utility 9D (CHU-9D), EuroQoL five-dimension youth version three-level (EQ-5D-Y-3L), short-form six-dimension (SF-6D), and Health Utilities Index (HUI) (9, 10). However, Pediatric Quality of Life Inventory^TM^ 4.0 (Peds QL 4.0) is often used in clinical studies of FD (11–13). It cannot directly measure HUVs (14) as a non-preference-based scale. To make full use of existing research data and obtain HUVs of FD patients, constructing a mapping algorithm from Peds QL 4.0 to preference-based scale is necessary. The CHU-9D, a multi-attribute utility scale for children and adolescents, is used worldwide (9, 11) and has been demonstrated by previous studies to have good psychometric properties in the Chinese population (15). Unlike other preference-based scales that apply to children and adolescents, CHU-9D was originally developed for young people (16). In addition, both the Peds QL 4.0 and CHU-9D had items to investigate the physical and psychosocial health of children and adolescents. For instance, the CHU-9D sad and sleep dimensions were captured in Peds QL 4.0 emotional functional dimension, and the pain and tired dimensions were captured in physical functioning dimension. The conceptual overlap makes it meaningful to develop a mapping algorithm between Peds QL 4.0 and CHU-9D.

Up to now, mapping has been regarded as the “second-best solution” for measuring HUVs. ISPOR has issued relevant guidance to guide researchers to use this method (17, 18). A large number of studies have shown that the mapping algorithm between Peds QL 4.0 and preference-based scale can be developed to obtain the HUV (19–24). However, no such study has been conducted for developing a mapping algorithm between Peds QL 4.0 and CHU-9D in Chinese FD children and adolescents. Thus, this study was to develop an algorithm based on Chinese FD patients. In addition, the mapping algorithm enables researchers to calculate HUVs using available clinical data, which facilitates the development of health technology evaluations.

## Methods

From March to May 2020, FD outpatients were recruited from several hospitals in Zhejiang province through convenient sampling. Meanwhile, we collected data related to the HRQoL of participants by CHU-9D and Peds QL 4.0. All participants voluntarily participated and signed informed consent. This study was approved by the Ethics Committee of China Pharmaceutical University.

### Sample

Inclusion criteria were as follows: (1) informed and voluntary; (2) age 6 to 17 years [the CHU-9D is suitable for children and adolescents aged 7–17 years. Studies have shown that it is also suitable for children aged 6–7 years (25)]; and (3) diagnosed with FD according to the Rome IV criteria (26). The exclusion criteria were as follows: (1) non-Chinese; (2) mental patients, unconsciousness, unable to describe their own situation.

### Data collection

Trained investigators went to hospitals to present the study to FD patients and their guardians and asked them about their willingness to participate. For patients who want to take part, investigators will provide them with an informed consent form and a questionnaire. After signing the forms in a quiet environment, they completed questionnaires in full view of investigators. The questionnaire includes two HRQoL tools. In addition, to avoid ranking bias, participants decided the order of filling by flipping a coin. Considering that younger patients may have difficulty in understanding the scale, the children younger than 7 years old completed the questionnaires with their guardians, while other participants completed on their own. For the basic information part of the questionnaire, the guardian should assist the participants to complete it. After the questionnaire was completed, the investigator would check the questionnaire and upload the data after the respondents confirmed the questions. After that, the auditor would review it again. For the questionnaire with obvious problems, the auditor would return the questionnaire.

### Questionnaire

The literature and experts' opinions were drawn upon (11, 13, 14, 27, 28). The questionnaire was divided into two parts as follows: basic information and health status. According to the results of the pilot survey in hospitals, we modified the questionnaire and formed the final version. Its rationality, readability, and comprehensibility were affirmed by experts and supported by the results of pilot research.

There were two parts to the questionnaires. Part 1 collected sociodemographic information, including age, gender, parents' education level, and family income. Part 2 collected some health status indicators reported by patients through CHU-9D and Peds QL 4.0.

### Child health utility 9D

Child Health Utility 9D (CHU-9D) was a universal scale developed for children and adolescents by Professor Katherine Stevens from the University of Sheffield. It was used to obtain children's and adolescents' HUVs of subjects and had been widely used at present. The translation process of the Chinese version of the CHU-9D questionnaire was designed based on the recommendations of the ISPOR Task Force (29). In 2013, a pilot study was conducted in Jiangsu province, China. Its results supported the feasibility and construct validity of the Chinese version of CHU-9D for measuring and valuing the HRQoL of Chinese young people (15). The CHU-9D consisted of nine dimensions that are “worried,” “sad,” “pain,” “tired,” “annoyed,” “schoolwork/homework,” “sleep,” “daily routine,” and “activities,” each dimension had five levels, a total of 5^9^ = 1,953,125 possible health states were defined. The CHU-9D was originally developed for children aged 7–11 years (16), but subsequent studies have shown that it can also be used for children aged 6 years and adolescents aged 11–17 years (25, 30). In this study, the CHU-9D utility scores were calculated using the China value set (27).

### Peds QL 4.0

Pediatric Quality of Life Inventory™ 4.0 (Peds QL 4.0) was developed under the lead of Professor Varni et al. (14) and was officially released in 1999. Peds QL 4.0 was introduced in China in 2004. Yi-Yun et al. developed the Chinese version of Peds QL 4.0 based on the standard procedure of cross-cultural adaptation (translation–back translation–cultural adaptation–pre-test) to (31–33). This version was proved that it was applicable to Chinese children (14, 33–35). The self-report versions of the Peds QL 4.0 for young children (aged 5–7 years), children (aged 8–12 years), and teenagers (aged 13–18 years) and parent-report versions of the Peds QL 4.0 for young children (aged 5–7 years) were used in the study. Peds QL 4.0 contains four dimensions as follows: physical functioning (PF), emotional functioning (EF), social functioning (SF), and school functioning (ScF). There were 23 items, and the recall period was 1 month. PF contained eight items, while EF, SF, and ScF each contained five items. Items were scored on a five-point Likert scale as follows: 0 indicates “never a problem,” 1 indicates “almost never a problem,” 2 indicates “sometimes a problem,” 3 indicates “often a problem,” and 4 indicates “almost always a problem.” Items were, then, transformed into a score ranging from 0 to 100 (where 0 = 100, 1 = 75, 2 = 50, 3 = 25, and 4 = 0). The dimension score was the average score of the items contained in the dimension, and the total score was the average score of all items responded.

### Data analysis

#### Descriptive statistics

Descriptive statistics [mean and standard deviation (SD) for continuous variables and frequency and percentage for categorical variables] were used for the sample characteristics. The distributions of the CHU-9D utility score and Peds QL 4.0 score were shown through Shapiro–Wilk test and figures.

#### Correlation test

Mapping of the scale requires some conceptual overlap between the initial scale and the target scale (36, 37). Spearman's rank correlations (ρ) were used in this study to test the conceptual overlap strength between CHU-9D and Peds QL 4.0. Conceptual overlap was characterized by the content similarity between HRQoL result measurements. If two scales lack conceptual overlap, the mapping relationship would not be established. In addition, we tested the correlation among the variables included in the study to ensure the low collinearity among the variables included in the mapping model. The strength of correlation could be divided into four levels (very weak = 0–0.19; weak = 0.20–0.39; moderate = 0.40–0.59; strong = 0.60–0.79; and very strong = 0.80–1.00) (38, 39).

#### Mapping model

Mapping consists of two broad approaches, such as direct mapping and response mapping. We used six regression models for developing a simpler and more accurate mapping algorithm, based on guidelines and previous research (17), including ordinary least squares regression (OLS), general linear regression model (GLM), MM-estimator model (MM), Tobit regression model (Tobit), Beta regression model (Beta) for direct mapping, and multinomial logistic regression (MLOGIT) for response mapping.

Ordinary least squares regression (OLS) uses linear functions to construct the relationship between independent variables and dependent variables. Due to its simplicity, OLS is widely used in direct mapping studies (38, 40). However, OLS performs poorly in predicting poor or full health, and the predicted values may be outside of the reasonable range (41–43). GLM (link “logit”), a flexible form of OLS, allows the outcome variables to have non-normal error distributions (44, 45). MM can better identify outliers, thus MM is less affected by outliers and has less deviation from the fitted residuals (23, 46). When using the traditional linear regression model, the predicted value is often out of the range of the dependent variable. In view of the fact that the CHU-9D scores are deleted at the upper and lower limits, OLS and GLM tend to produce systematic bias. We also used the Tobit model and Beta model. As a censored model, the Tobit model can be used to predict the continuous but limited or truncated dependent variables, but it is more sensitive to heteroscedasticity and non-normal distribution (47, 48). The Beta model solves this problem by assuming that the value of the dependent variable is between 0 and 1, and the model is also suitable for cases with heteroscedasticity or non-normal distribution of the data (49, 50). In response mapping, we used the MLOGIT model to obtain the probability of a specific level of CHU-9D in each dimension and then calculated the utility value of CHU-9D using the expected utility value method (50).

#### Variables

We chose the CHU-9D total score and scores of each dimension of CHU-9D as the dependent variable for direct and response mapping, and Peds QL 4.0 total score, Peds QL 4.0 dimension scores, and Peds QL 4.0 item scores were used as independent variables for regression to generate mapping algorithm. In addition, to ensure the accuracy of the mapping algorithm, age and gender were included as independent variables according to the correlation between variables. Finally, six combination models of independent variables were developed, as presented in Table 1.

**Table 1:** Combinations of variables.

**Combination**	**Independent variables**
Combination 1	Peds QL 4.0 total score
Combination 2	Peds QL 4.0 total score, age, gender
Combination 3	Peds QL 4.0 dimension scores
Combination 4	Peds QL 4.0 dimension scores, age, gender
Combination 5	Peds QL 4.0 item scores^a^
Combination 6	Peds QL 4.0 item scores^a^, age, gender

a Peds QL 4.0 items were selected according to statistical significance using stepwise regression, and the statistical significance level was 0.05. Variables eventually included in the study: PF2, Hard to run; PF5, Hard to take a bath or shower; PF8, Low energy; EF1, Feel afraid or scared; EF4, Trouble sleeping; EF5, Worry about what will happen; SF1, Trouble getting along with others; SF3, Teased; SF4, Not able to do things that others can do; ScF1, Hard to pay attention in class; ScF2, Forget things; ScF3, Trouble keeping up with schoolwork; ScF4, Miss school because of not feeling well.

#### Validation and comparison of mapping algorithms

The 10-fold cross-validation method was used to predict model performance. In this method, the original sample was divided into 10 subsamples of roughly equal size. In total. One of the 10 subsamples was taken as the validation sample, and the remaining subsamples were taken as the training samples for regression (23, 45).

The mean of the HUVs predicted (mean P), root mean square error (RMSE), mean absolute error (MAE), adjusted R^2^ (adj R^2^), and concordance correlation coefficient (CCC) was recorded and averaged for each combination. The mapping algorithm with the best comprehensive ranking was the optimal mapping algorithm in different combinations of variables (18, 45).

All statistical analyses were performed by stata15, programs R and Microsoft^®^ Excel 2016.

## Result

### Participant characteristics

**Figure 1:**
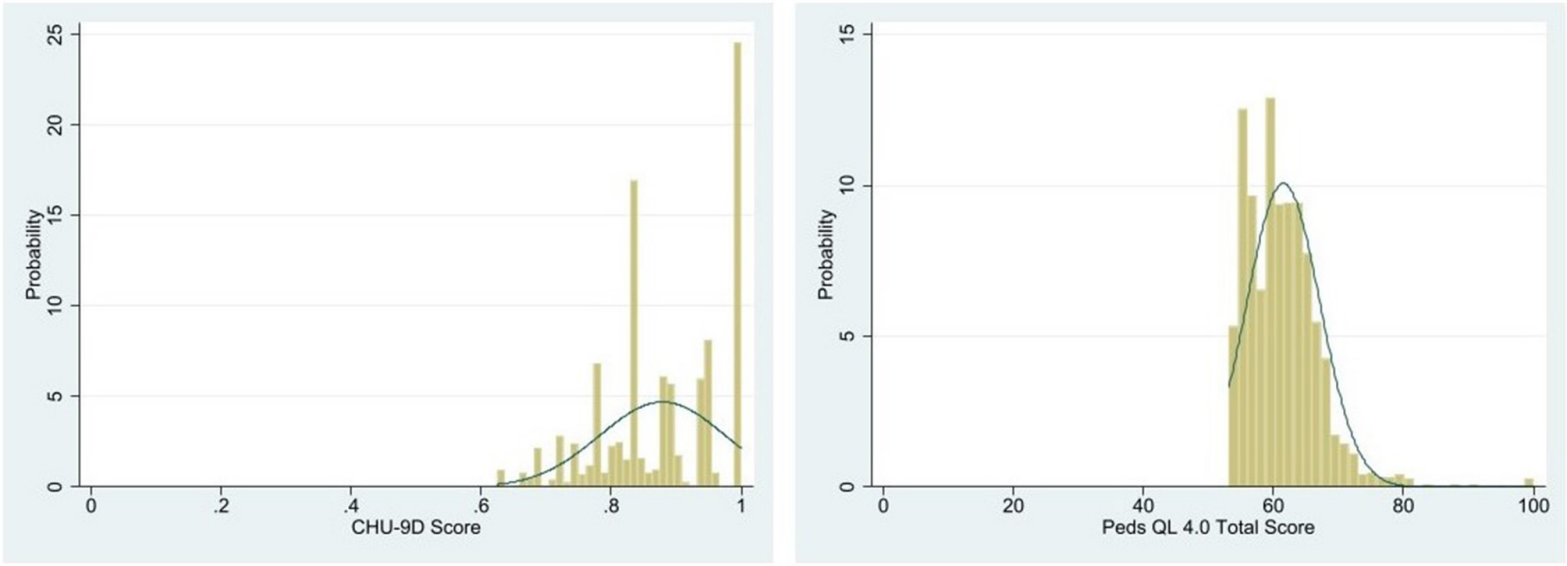
Distributions of CHU-9D utility score and Peds QL 4.0 total score.

A total of 2,152 eligible FD patients were enrolled in this study (Table 2), of whom 1,155 patients had their guardians complete the questionnaire on their behalf. Their mean age (SD) was 7.23 (1.47) years, the mean utility score of CHU-9D (SD) was 0.88 (0.10), and the mean score of Peds QL 4.0 (SD) was 61.52 (5.63). Participants' CHU-9D utility score and Peds QL 4.0 total score were skewed (Figure 1).

**Table 2:** Participant characteristics.

**Characteristics (*N =* 2,152)**	**Mean ±SD/N (%)**	**Median**	**Min**	**Max**
Age (years)	7.23 ± 1.47	6	6	17
**Gender**				
Boys	1,177 (54.69%)			
Girls	975 (45.31%)			
CHU-9D	0.88 ± 0.10	0.88	0.62	1.00
Peds QL 4.0 total score	61.52 ± 5.63	60.87	53.12	100.00
Peds QL 4.0 Physical Functioning	67.02 ± 5.49	62.50	50.00	100.00
Peds QL 4.0 Emotional Functioning	57.45 ± 5.87	55.00	40.00	100.00
Peds QL 4.0 Social Functioning	57.21 ± 10.09	55.00	40.00	100.00
Peds QL 4.0 School Functioning	62.26 ± 6.46	60.00	40.00	100.00

SD, standard deviation.

### Correlation test results

Correlations between CHU-9D utility score, Peds QL 4.0 total score, Peds QL 4.0 dimension scores, Peds QL 4.0 item scores, age, and gender are presented in Appendix 1. There was a strong positive correlation between the CHU-9D utility score and Peds QL 4.0 total score (ρ = 0.6836, *p* < 0.001), as well as the Peds QL 4.0 dimension scores and the CHU-9D utility score, among which the SF dimension had the highest correlation (ρ = 0.6399, *p* < 0.001) and the PF dimension had the lowest correlation (ρ = 0.3146, *p* < 0.001).

### Performance of mapping algorithms

The results of mapping algorithm performance based on six regression techniques are presented in Table 3.

**Table 3:** Goodness-of-fit results from the full estimation sample (*N* = 2,152).

**Model**	**Mean P (SD)**	**Min P**	**Max P**	**MAE**	**RMSE**	**Adjr^2^**	**CCC**
**Combination 1**							
OLS	0.8787 (0.0551)	0.7966	1.2554	0.0641	0.0794	0.3246	0.4905
GLM	0.8789 (0.0504)	0.8095	1.2709	0.0650	0.0810	0.2973	0.4478
MM	0.8841 (0.0661)	0.7854	1.3366	0.0641	0.0803	0.3084	0.5304
TOBIT	0.8767 (0.0473)	0.7966	1.0000	0.0621	0.0770	0.3646	0.4877
BETA	0.8862 (0.0678)	0.7113	0.9998	**0.0576**	0.0719	0.4465	**0.6308**
MLOGIT	0.8787 (0.0603)	0.7751	0.9987	0.0580	**0.0716**	**0.4498**	0.6045
**Combination 2**							
OLS	0.8787 (0.0573)	0.7968	1.3113	0.0617	0.0778	0.3516	0.5212
GLM	0.8789 (0.0532)	0.8085	1.3626	0.0624	0.0792	0.3269	0.4844
MM	0.8832 (0.0674)	0.7867	1.3904	0.0616	0.0786	0.3385	0.5564
TOBIT	0.8771 (0.0511)	0.7968	1.0000	0.0602	0.0757	0.3862	0.5211
BETA	0.8861 (0.0679)	0.7303	0.9999	**0.0572**	**0.0716**	**0.4504**	**0.6342**
MLOGIT	0.8787 (0.0617)	0.7763	0.9991	0.0577	0.0719	0.4465	0.6073
**Combination 3**							
OLS	0.8787 (0.0613)	0.7615	1.1514	0.0604	0.0747	0.4023	0.5749
GLM	0.8788 (0.059)	0.7730	1.1871	0.0610	0.0756	0.3869	0.5542
MM	0.8844 (0.0652)	0.7594	1.1799	0.0597	0.0750	0.3969	0.5875
TOBIT	0.8774 (0.0576)	0.7615	1.0000	0.0591	0.0732	0.4250	0.5768
BETA	0.8844 (0.0783)	0.6222	0.9987	0.0584	0.0735	0.4211	**0.6522**
MLOGIT	0.8787 (0.0636)	0.7610	0.9981	**0.0559**	**0.0692**	**0.4863**	0.6424
**Combination 4**							
OLS	0.8787 (0.0647)	0.7616	1.2422	0.0577	0.0718	0.4478	0.6200
GLM	0.8788 (0.0625)	0.7722	1.2736	0.0584	0.0728	0.4327	0.6012
MM	0.884 (0.0682)	0.7619	1.2772	0.0572	0.0721	0.4431	0.6302
TOBIT	0.8776 (0.0614)	0.7616	1.0000	0.0566	0.0705	0.4671	0.6214
BETA	0.8839 (0.0809)	0.6482	0.9996	0.0575	0.0725	0.4364	**0.6704**
MLOGIT	0.8787 (0.067)	0.7591	0.9994	**0.0543**	**0.0677**	**0.5093**	0.6695
**Combination 5**							
OLS	0.8787 (0.0699)	0.7471	1.1365	0.0530	0.0669	0.5207	0.6873
GLM	0.8788 (0.0686)	0.7566	1.1429	0.0534	0.0675	0.5114	0.6773
MM	0.8853 (0.0718)	0.7485	1.1651	0.0520	0.0673	0.5140	0.6899
TOBIT	0.878 (0.0682)	0.7471	1.0000	0.0523	0.0661	0.5318	0.6893
BETA	0.8832 (0.0852)	0.6559	0.9994	0.0529	0.0676	0.5100	**0.7265**
MLOGIT	0.8787 (0.0711)	0.7405	0.9994	**0.0502**	**0.0633**	**0.5709**	0.7235
**Combination 6**							
OLS	0.8787 (0.0702)	0.7548	1.1417	0.0529	0.0665	0.5256	0.6919
GLM	0.8788 (0.069)	0.7644	1.1488	0.0533	0.0672	0.5162	0.6818
MM	0.8852 (0.0718)	0.7511	1.1649	0.0519	0.0670	0.5189	0.6931
TOBIT	0.878 (0.0685)	0.7548	1.0000	0.0521	0.0658	0.5358	0.6934
BETA	0.8831 (0.0853)	0.6608	0.9994	0.0525	0.0670	0.5188	**0.7318**
MLOGIT	0.8787 (0.0715)	0.7420	0.9995	**0.0501**	**0.0630**	**0.5745**	0.7271

Numbers in bold were the best values in each combination. OLS, Ordinary least squares regression; GLM, the general linear regression model; MM, MM-estimator model; Tobit, Tobit regression model; Beta, Beta regression model; MLOGIT, multinomial logistic regression. Mean P, mean of the health utility score predicted; Min P, minimum of the health utility score predicted; Max P, maximum of the health utility score predicted; MAE, mean absolute error; RMSE, root mean square error; adjR^2^, adjusted R^2^; CCC, concordance correlation coefficient.

For model combinations based on the full estimation sample, the mean P ranged from 0.8767 (Tobit of Combination 1) to 0.8862 (Beta of Combination 1), with the OLS of Combination 3 having the closest predicted score (0.8784) to the mean score. In Combination 1, Beta had the lowest MAE (0.0576), while MLOGIT performed better in RMSE (0.0716) and adj R^2^ (0.4498). In Combination 2, Beta performed better than other models. In Combinations 3 to 6, MLOGIT had the lowest comprehensive ranking and better performance than other models. The Beta had the best CCC in all combinations. Among all combinations, MLOGIT in Combination 6 had the best MAE (0.0501), RMSE (0.0630), and adj R^2^ (0.5745), and the Beta model had the best CCC (0.7319). In summary, the performance based on Peds QL 4.0 item scores (Combinations 5 and 6) was better than other combinations. MLOGIT had better performance in the validation index, with the best MAE, RMSE, and adj R^2^, followed by the Beta model and Tobit model, and the difference between MLOGIT and Beta was very small.

### Validation

The results showed that MLOGIT had the best performance. However, the absence of some dimension levels may lead to abnormal or biased fitting results of MLOGIT. Moreover, there was a small difference between MLOGIT and the second-best model in each index. Thus, we preliminarily concluded that choosing the second-best model would be more helpful to obtain accurate results. MLOGIT was not validated in this study.

Table 4 summarizes the validation results of the model through the 10-fold cross-validation method. In all combinations, Beta and Tobit performed better on the validation index. In Combinations 1 to 2, Beta had the lowest ranking and better performance than other models. In Combination 3, MAE and CCC of Beta were superior to Tobit, while RMSE and adj R^2^ of Tobit were superior. The comprehensive ranking of the two models was consistent. Considering that RMSE was more sensitive to potential outliers, more weight could be given to MAE in this case (51). Therefore, we concluded that Beta was better in this combination. In Combinations 4 to 6, the Tobit had the lowest comprehensive ranking and better performance than other models. Of all the combinations, Tobit had the best MAE (0.0559), RMSE (0.0685), and adj R^2^ (0.4973) in Combination 5, and Beta had the best CCC (0.7278) in Combination 6.

**Table 4:** Goodness-of-fit results from the 10-fold cross-validation.

**Model**	**Mean P (SD)**	**Min P**	**Max P**	**MAE**	**RMSE**	**Adj R^2^**	**CCC**
**Combination 1**							
OLS	0.8788 (0.0023)	0.8027	1.1816	0.0647	0.0798	0.3200	0.4889
GLM	0.8789 (0.0022)	0.8143	1.1874	0.0656	0.0814	0.2919	0.4460
MM	0.8841 (0.0028)	0.7928	1.2471	0.0647	0.0806	0.3042	0.5291
TOBIT	0.8767 (0.0019)	0.8027	1.0000	0.0626	0.0773	0.3615	0.4871
BETA	0.8862 (0.0034)	0.7317	0.9982	**0.0581**	**0.0721**	**0.4435**	**0.6300**
**Combination 2**							
OLS	0.8787 (0.003)	0.7991	1.1785	0.0629	0.0785	0.3403	0.5190
GLM	0.8789 (0.003)	0.8099	1.1903	0.0636	0.0801	0.3139	0.4820
MM	0.8832 (0.0035)	0.7897	1.2349	0.0628	0.0792	0.3279	0.5547
TOBIT	0.8771 (0.0026)	0.7991	1.0000	0.0613	0.0763	0.3769	0.5197
BETA	0.886 (0.0035)	0.7360	0.9981	**0.0583**	**0.0722**	**0.4416**	**0.6326**
**Combination 3**							
OLS	0.8788 (0.0031)	0.7849	1.1086	0.0618	0.0756	0.3885	0.5733
GLM	0.8789 (0.003)	0.7926	1.1157	0.0625	0.0766	0.3724	0.5527
MM	0.8845 (0.0034)	0.7835	1.1266	0.0611	0.0759	0.3828	0.5860
TOBIT	0.8774 (0.0031)	0.7849	1.0000	0.0604	**0.0741**	**0.4133**	0.5758
BETA	0.8843 (0.0042)	0.7025	0.9968	**0.0597**	0.0743	0.4092	**0.6507**
**Combination 4**							
OLS	0.8788 (0.0034)	0.7802	1.1206	0.0597	0.0731	0.4278	0.6176
GLM	0.8789 (0.0033)	0.7881	1.1356	0.0604	0.0742	0.4103	0.5983
MM	0.8884 (0.0101)	0.7841	1.1459	0.0592	0.0744	0.4065	0.6229
TOBIT	0.8775 (0.0035)	0.7802	1.0000	**0.0584**	**0.0717**	**0.4504**	0.6196
BETA	0.8838 (0.0041)	0.6964	0.9959	0.0593	0.0736	0.4194	**0.6686**
**Combination 5**							
OLS	0.8788 (0.004)	0.7608	1.0938	0.0568	0.0694	0.4833	0.6826
GLM	0.8789 (0.0039)	0.7681	1.1022	0.0572	0.0701	0.4730	0.6725
MM	0.8893 (0.0088)	0.7705	1.1301	0.0560	0.0712	0.4557	0.6739
TOBIT	0.878 (0.0038)	0.7608	1.0000	**0.0559**	**0.0685**	**0.4973**	0.6852
BETA	0.8832 (0.0049)	0.6937	0.9979	0.0566	0.0700	0.4744	**0.7229**
**Combination 6**							
OLS	0.8788 (0.0040)	0.7633	1.0937	0.0572	0.0695	0.4824	0.6865
GLM	0.8789 (0.0039)	0.7705	1.1023	0.0577	0.0702	0.4716	0.6762
MM	0.8852 (0.0042)	0.7641	1.1107	0.0563	0.0699	0.4759	0.6884
TOBIT	0.8779 (0.0038)	0.7633	1.0000	**0.0563**	**0.0686**	**0.4962**	0.6887
BETA	0.8831 (0.0047)	0.7070	0.9975	0.0568	0.0698	0.4782	**0.7278**

Numbers in bold were the best values in each combination. OLS, Ordinary least squares regression; GLM, the general linear regression model; MM, MM-estimator model; Tobit, Tobit regression model; Beta, Beta regression model; MLOGIT, multinomial logistic regression. Mean P, mean of the health utility score predicted; Min P, minimum of the health utility score predicted; Max P, maximum of the health utility score predicted; MAE, mean absolute error; RMSE, root mean square error; adjR^2^, adjusted R^2^; CCC, concordance correlation coefficient.

### Best-performing mapping algorithm

**Figure 2:**
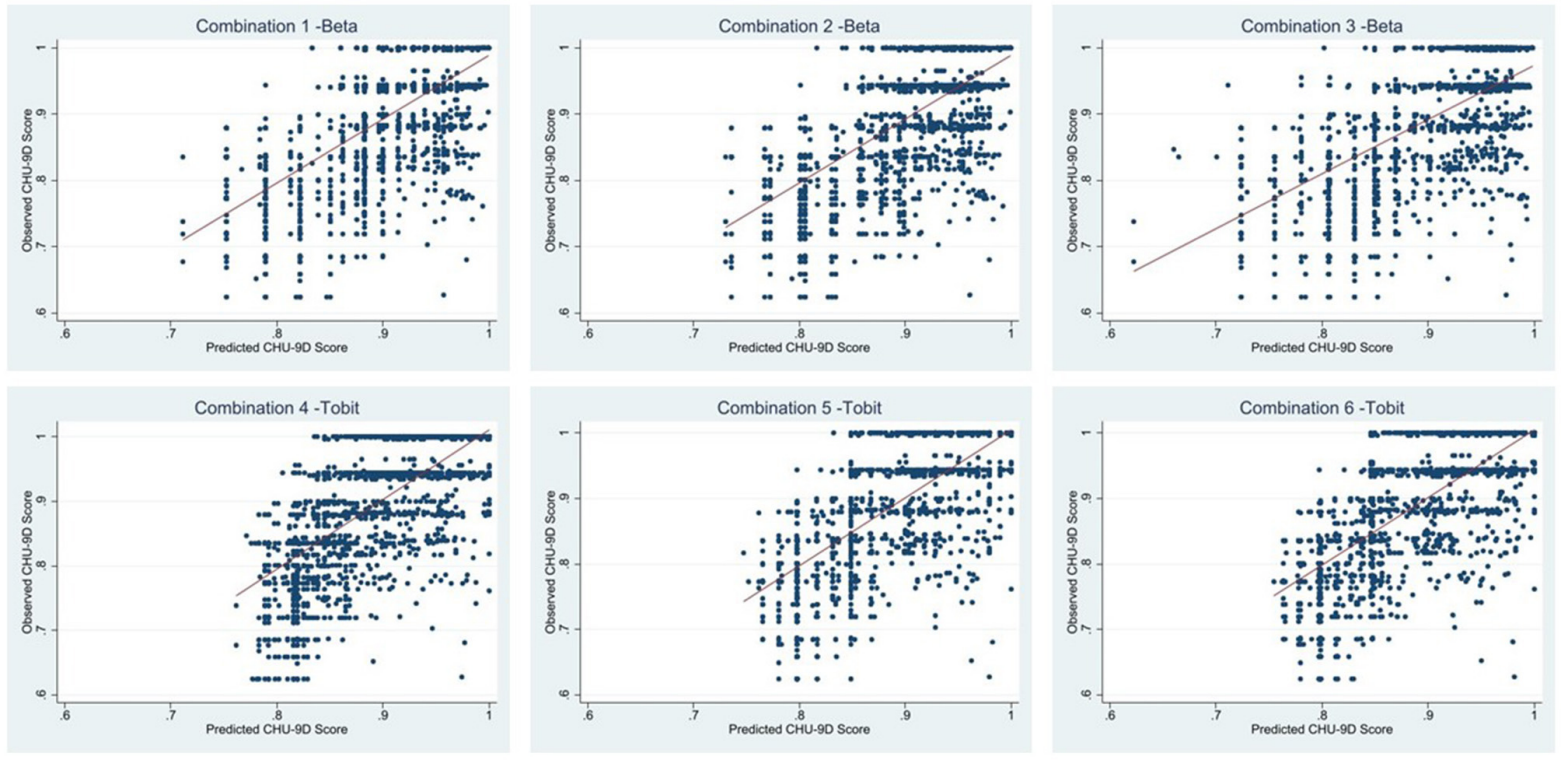
The observed and predicted CHU-9D utility score.

According to the comprehensive ranking of the four indexes, the Tobit with Peds QL 4.0 item scores, gender, and age as independent variables (Combination 6) was the best model to predict the CHU-9D utility score. However, the Peds QL 4.0 item scores were difficult to obtain in reality. Hence, we provided the parameters of the optimal mapping algorithm for the CHU-9D utility score of different combinations (Table 5) and the consistency between the predicted CHU-9D utility score and the observed CHU-9D utility score (Figure 2). The Pearson correlation coefficients were 0.6836, 0.6788, 0.6845, 0.7092, 0.7455, and 0.7476 for Combinations 1 to 6, respectively. They indicated a high correlation between the observed CHU-9D utility scores and predicted CHU-9D utility scores. On the basis of these results, we suggest that researchers choose different variable combinations and corresponding mapping algorithms depending on the available data.

**Table 5:** Regression outputs of the mapping model.

	**Combination1**	**Combination2**	**Combination3**	**Combination4**	**Combination5**	**Combination6**
Intercept	−7.9832 (0.2245)^***^	−7.6672 (0.2247)^***^	−4.0199 (0.2942)^***^	0.5415 (0.0263)^***^	0.8991 (0.0301)^***^	0.8652 (0.0313)^***^
Total score/100	16.7248 (0.3761)^***^	15.8034 (0.4217)^***^				
PF score/100			3.2633 (0.3777)^***^	−0.1221 (0.0465)^**^		
EF score/100			−5.5249 (0.4787)^***^	−0.2637 (0.0409)^***^		
SF score/100			9.2555 (0.3088)^***^	0.5415 (0.0259)^***^		
ScF score/100			3.3013 (0.4243)^***^	0.1888 (0.0373)^***^		
PF2/100					−0.1566 (0.0283)^***^	−0.1721 (0.0283)^***^
PF5/100					0.2031 (0.0123)^***^	0.1234 (0.0209)^***^
PF8/100					−0.1554 (0.0339)^***^	−0.1491 (0.0339)^***^
EF1/100					−0.1134 (0.0221)^***^	−0.0996 (0.0222)^***^
EF4/100					−0.0721 (0.0236)^**^	−0.0479 (0.0240)^*^
EF5/100					0.0423 (0.0176)^*^	0.0338 (0.0175)
SF1/100					0.0605 (0.0162)^***^	0.0581 (0.0162)^***^
SF3/100					0.2412 (0.0188)^***^	0.2432 (0.0187)^***^
SF4/100					0.1554 (0.0209)^***^	0.1617 (0.0208)^***^
ScF1/100					0.1453 (0.0120)^***^	0.1403 (0.0120)^***^
ScF2/100					0.0755 (0.0134)^***^	0.064 (0.0135)^***^
ScF3/100					−0.1907 (0.0360)^***^	−0.1996 (0.0360)^***^
ScF4/100					−0.1386 (0.0349)^***^	−0.1218 (0.0349)^***^
Female		−0.1698 (0.0387)^***^		−0.0058 (0.0033)		0.0016 (0.0030)
Age		0.0446 (0.0155)^**^		0.0202 (0.0016)^***^		0.0089 (0.0019)^***^

* p < 0.1,

** p < 0.05,

*** p < 0.01.

The Peds QL 4.0 scores used here should be the scaled scores (divided by 100). Total score, Peds QL 4.0 Total score; PF, Physical Functioning; EF, Emotional Functioning; SF, Social Functioning; ScF, School Functioning. PF2, Hard to run; PF5, Hard to take a bath or shower; PF8, Low energy; EF1, Feel afraid or scared; EF4, Trouble sleeping; EF5, Worry about what will happen; SF1, Trouble getting along with others; SF3, Teased; SF4, Not able to do things that others can do; ScF1, Hard to pay attention in class; ScF2, Forget things; ScF3, Trouble keeping up with schoolwork; ScF4, Miss school because of not feeling well.

### Mapping algorithm

Based on the conclusions from the section best-performing mapping algorithm, the mapping algorithm formula can be shown as follows:

Combination 1–3: Beta Model.


$$\begin{array}{l} Beta\;Prediction=Intercept+\beta 1*X1+\beta 2*X2\ldots +\beta i*Xi\\ \;CHU-9D\;Prediction=exp(Beta\;Prediction)/\\ \;(1+exp(Beta\;Prediction)) \end{array}$$

Combination 4–6: Tobit Model.


$$\begin{array}{l} y=Intercept+\beta 1*X1+\beta 2*X2\ldots +\beta i*Xi\\ \;CHU-9DPrediction=\{\begin{array}{c} 1\;ify>1\\ y\;otherwise \end{array} \end{array}$$

[X_i_ is the independent variable, such as total Score, gender, and age. β_i_ is the coefficient (parameters are presented in Table 4)].

## Discussion

Up to now, three types of research have constructed mapping algorithms from Peds QL 4.0 to CHU-9D based on children and adolescents (19–22), but none of them are based on the Chinese population. Considering the differences and uniqueness of populations in different countries, this study is the first to construct a mapping algorithm from Peds QL 4.0 to CHU-9D based on Chinese children and adolescent FD patients. This mapping algorithm is able to get HUVs by converting the non-preference-based Peds QL 4.0 into preference-based CHU-9D scores. It can improve both the efficiency of available clinical data and help decision makers to compare and evaluate relevant interventions, facilitating the development of pharmacoeconomic evaluation.

In accordance with guidance (17), we used six regression models to develop mapping algorithms from Peds QL 4.0 to CHU-9D, including OLS, GLM, MM, Tobit, Beta, and MLOGIT, among which Tobit performed best, followed by Beta. Some studies indicated that indirect mapping could improve the fitting of models compared with direct mapping using linear models (52). In this study, although the sample size was large and the MLOGIT performance was good, we still believed that Tobit or Beta was better. This was mainly due to the lack of dimension level of CHU-9D, which leads to the concentration in some health states. This may lead to an offset in the final result of MLOGIT. In addition, we also found that it could easily lead to fitting abnormalities in MLOGIT fitting process. Therefore, our final results did not use the MLOGIT due to the fact that it requires more comprehensive health state data to prove its accuracy. Furthermore, previous studies had shown that MM was superior to MLOGIT, which was different from our results (19).

In the existing mapping research of Peds QL 4.0, the Peds QL 4.0 total score, the Peds QL 4.0 dimension scores, and the Peds QL 4.0 item scores were often used as independent variables (19–21, 24), as well as some demographic indicators such as age and gender. We screened the independent variables according to the correlation between the variables (see Appendix 1). Some variables that were highly correlated with CHU-9D utility score were directly included as independent variables, for instance, Peds QL 4.0 total score (0.6836), SF (0.6399), and ScF (0.5066). To improve the prediction ability of the model as much as possible, we also screened the variables with low correlation according to the independent variables included in previous studies, such as trying to include gender as an independent variable. In addition, we tried to use the Peds QL 4.0 item scores as independent variables. Due to the large number of Peds QL 4.0 item scores, in order to avoid excessive complexity of the model, we used the stepwise regression method to select Peds QL 4.0 item scores that had a significant correlation with CHU-9D utility value. Finally, the Peds QL 4.0 total score, Peds QL 4.0 dimension scores, Peds QL 4.0 item scores, gender, and age were used as independent variables. We found that when age and gender were used as independent variables, the 10-fold cross-validation and the fitting of the full estimation sample showed different results. In the full estimation sample, the inclusion of age and gender improved the performance of the model. However, in the 10-fold cross-validation, only the model performance of the combination of independent variables based on the Peds QL 4.0 dimension scores was improved. The result was similar to Tosin's research (20). Considering the obvious influence of gender and age on the population HUVs, we propose that it is necessary to include age and gender in the construction of the mapping algorithm, but more research is needed to prove the difference.

This study evaluated the prediction performance of different models in different combinations based on full samples and ranked the model performance based on MAE, RMSE, adj R^2^, and CCC using the 10-fold cross-validation method. Similar to several other studies, almost all the estimators overestimated the lower bound of the CHU-9D utility score and overpredicted the upper bound of 1 (21, 22). Although overprediction is a difficult problem, some studies have shown that it can be dealt with by truncating or setting the value of the dependent variable between 0 and 1 to the boundary value, such as Tobit model and Beta model (24, 53, 54). Overall, the predictive performance of the Beta model and Tobit model was consistent with the MAE range (0.0408–0.1270) and RMSE range (0.0594–0.1619) observed in the CHU-9D mapping study (19–23).

There are also some limitations in the study. First, although the sample size was large, the sample source was limited to Zhejiang province, which was difficult to represent the level of China. Second, we used the 10-fold cross-validation method to verify the mapping algorithm, but no external validation was performed due to the lack of available data. Third, in the process of data sampling and collection, many samples are concentrated in the lower age group (average age = 7.23), and nearly half of the questionnaires were filled by guardians, which may be related to the factors that children in the lower age group are more likely to receive attention and seek medical treatment in time. However, guardians could hard to understand the real feelings of young people, which may cause agency bias. Fourth, there were some missing CHU-9D dimension levels in the study sample, which may lead to the limited prediction range of the mapping algorithm and the deviation of indirect mapping results (17). Therefore, the model based on indirect mapping was not recommended in the study, and better data were needed to verify in future.

## Conclusion

The research first developed a mapping algorithm from Peds QL 4.0 to CHU-9D based on Chinese children and adolescents. We also constructed different mapping algorithms for different combinations of variables, and of all algorithms, the Tobit model with Peds QL 4.0 item scores, gender, and age as the independent variables was the most accurate. However, researchers can reasonably choose mapping algorithms for different combinations of variables based on available data to conduct other studies such as pharmacoeconomic evaluations and provide references for relevant policymakers in China.

## Data availability statement

The raw data supporting the conclusions of this article will be made available by the authors, without undue reservation.

## Author contributions

QW, CW, and XX made their contributions to the conception and design of the study. QW, YH, and CW made their contributions to the acquisition and analysis of the data. ML and YH made their contributions to the interpretation of data. QW made contributions to the drafting of the study. QW, ML, and XX made their contributions to the revision of the study. All authors of this study has approved the submitted version and has agreed both to be personally accountable for the author's own contributions and ensure that questions related to the accuracy or integrity of any part of the study.
