# Supplementary material for: Mapping the Peds QLTM 4.0 onto CHU-9D: a cross-sectional study in functional dyspepsia population from China
Source: Front Public Health. 2023 May 31;11:1166760. doi: 10.3389/fpubh.2023.1166760 (PMC10266104; doi:10.3389/fpubh.2023.1166760)
Supplement: Supplementary file 1 [file Table_1.DOCX]

Appendix 1a The Spearman's correlation coefficient between variables

| Variables | CHU-9D | Gender | Age | Total score | PF | EF | SF | ScF | PF1 | PF2 | PF3 | PF4 | PF5 | PF6 | PF7 |
| --- | --- | --- | --- | --- | --- | --- | --- | --- | --- | --- | --- | --- | --- | --- | --- |
| CHU-9D | 1 |  |  |  |  |  |  |  |  |  |  |  |  |  |  |
| Gender | -0.0450** | 1 |  |  |  |  |  |  |  |  |  |  |  |  |  |
| Age | 0.3983*** | -0.0935*** | 1 |  |  |  |  |  |  |  |  |  |  |  |  |
| Total score | 0.6836*** | 0.0140 | 0.5086*** | 1 |  |  |  |  |  |  |  |  |  |  |  |
| PF | 0.3146*** | -0.0634*** | 0.7208*** | 0.5958*** | 1 |  |  |  |  |  |  |  |  |  |  |
| EF | 0.3644*** | -0.0031 | 0.1328*** | 0.6665*** | 0.294*** | 1 |  |  |  |  |  |  |  |  |  |
| SF | 0.6399*** | -0.0385* | 0.2206*** | 0.8645*** | 0.2547*** | 0.5920*** | 1 |  |  |  |  |  |  |  |  |
| ScF | 0.5066*** | 0.1822*** | 0.1126*** | 0.7419*** | 0.1661*** | 0.4524*** | 0.6743*** | 1 |  |  |  |  |  |  |  |
| PF1 | 0.1332*** | 0.0805*** | 0.1184*** | 0.2131*** | 0.1693*** | 0.2204*** | 0.2336*** | 0.1365*** | 1 |  |  |  |  |  |  |
| PF2 | -0.0772*** | -0.0039 | 0.1783*** | 0.0586*** | 0.3955*** | 0.0535** | -0.0584*** | -0.0024 | 0.3117*** | 1 |  |  |  |  |  |
| PF3 | 0.3867*** | -0.0691*** | 0.8018*** | 0.617*** | 0.9316*** | 0.2733*** | 0.2855*** | 0.1533*** | 0.1282*** | 0.2297*** | 1 |  |  |  |  |
| PF4 | 0.3618*** | -0.0786*** | 0.7872*** | 0.5851*** | 0.9194*** | 0.2395*** | 0.2480*** | 0.1291*** | 0.0936*** | 0.2112*** | 0.9589*** | 1 |  |  |  |
| PF5 | 0.3935*** | -0.0710*** | 0.8278*** | 0.6071*** | 0.9290*** | 0.2492*** | 0.2754*** | 0.1447*** | 0.1830*** | 0.2041*** | 0.9532*** | 0.9394*** | 1 |  |  |
| PF6 | -0.1572*** | 0.0141 | -0.1600*** | -0.1893*** | -0.0164 | -0.1183*** | -0.2176*** | -0.0888*** | -0.0807*** | 0.0013 | -0.2031*** | -0.1624*** | -0.1505*** | 1 |  |
| PF7 | 0.1880*** | 0.0494** | -0.0204 | 0.3184*** | 0.2821*** | 0.3608*** | 0.3351*** | 0.2430*** | 0.4011*** | 0.1678*** | 0.1741*** | 0.1247*** | 0.1815*** | 0.0225 | 1 |
| PF8 | 0.0368* | -0.0314 | -0.0372* | 0.2541*** | 0.2924*** | 0.3127*** | 0.2266*** | 0.2182*** | 0.1053*** | 0.1962*** | 0.2086*** | 0.1906*** | 0.1343*** | -0.0971*** | 0.0985*** |
| EF1 | 0.2303*** | 0.0452** | 0.0647*** | 0.4921*** | 0.2081*** | 0.7004*** | 0.4350*** | 0.3570*** | 0.2840*** | 0.1453*** | 0.1535*** | 0.1322*** | 0.1522*** | 0.0105 | 0.4495*** |
| EF2 | 0.1834*** | -0.0000 | 0.0302 | 0.4085*** | 0.1792*** | 0.5954*** | 0.3414*** | 0.3167*** | 0.1853*** | 0.1521*** | 0.1064*** | 0.0838*** | 0.1096*** | 0.1338*** | 0.4587*** |
| EF3 | 0.1802*** | -0.0034 | 0.0257 | 0.4053*** | 0.1699*** | 0.5910*** | 0.3413*** | 0.3204*** | 0.1859*** | 0.1445*** | 0.0921*** | 0.0753*** | 0.1050*** | 0.1437*** | 0.4379*** |
| EF4 | 0.0506** | -0.0030 | -0.1204*** | 0.1882*** | 0.2378*** | 0.2435*** | 0.2025*** | 0.1754*** | 0.0839*** | 0.0768*** | 0.1782*** | 0.1468*** | 0.1257*** | -0.0578*** | 0.3437*** |
| EF5 | 0.3245*** | -0.0084 | 0.2324*** | 0.4168*** | 0.1474*** | 0.6439*** | 0.3859*** | 0.2166*** | 0.2957*** | -0.0195 | 0.2083*** | 0.1743*** | 0.2096*** | -0.1588*** | 0.1526*** |
| SF1 | 0.5564*** | -0.0113 | 0.2047*** | 0.7216*** | 0.1860*** | 0.4731*** | 0.8261*** | 0.5771*** | 0.2203*** | -0.0011 | 0.2172*** | 0.1839*** | 0.2120*** | -0.1660*** | 0.2916*** |
| SF2 | 0.6093*** | -0.0564*** | 0.2273*** | 0.8094*** | 0.2634*** | 0.5686*** | 0.9048*** | 0.6329*** | 0.1896*** | -0.0687*** | 0.3000*** | 0.2610*** | 0.2843*** | -0.2152*** | 0.3099*** |
| SF3 | 0.6326*** | -0.0639*** | 0.2037*** | 0.8000*** | 0.2649*** | 0.6034*** | 0.8645*** | 0.6532*** | 0.1658*** | -0.0581*** | 0.2869*** | 0.2485*** | 0.2696*** | -0.2025*** | 0.3279*** |
| SF4 | 0.1615*** | 0.0558*** | 0.0093 | 0.2130*** | 0.0788*** | 0.1926*** | 0.2950*** | 0.1407*** | 0.3655*** | 0.0677*** | 0.0601*** | 0.0310 | 0.0886*** | -0.0192 | 0.3724*** |
| SF5 | 0.1520*** | 0.0775*** | 0.0443** | 0.2145*** | 0.0388* | 0.1861*** | 0.3166*** | 0.1130*** | 0.3499*** | 0.0730*** | 0.0409* | 0.0204 | 0.0679*** | -0.0450** | 0.2029*** |
| ScF1 | 0.5253*** | -0.0376* | 0.1430*** | 0.6289*** | 0.1446*** | 0.3731*** | 0.6001*** | 0.7669*** | 0.0614*** | 0.0248 | 0.1406*** | 0.1268*** | 0.1322*** | -0.0038 | 0.1736*** |
| ScF2 | 0.4390*** | -0.0395* | 0.2040*** | 0.5472*** | 0.0699*** | 0.3338*** | 0.5583*** | 0.6285*** | 0.1972*** | -0.0713*** | 0.1229*** | 0.0945*** | 0.1289*** | -0.1297*** | 0.1061*** |
| ScF3 | 0.1267*** | 0.0395* | 0.0946*** | 0.2090*** | 0.1349*** | 0.1917*** | 0.2286*** | 0.2246*** | 0.4855*** | 0.0800*** | 0.1082*** | 0.0728*** | 0.1529*** | 0.0541** | 0.3850*** |
| ScF4 | 0.1440*** | -0.0142 | -0.0448** | 0.4033*** | 0.3057*** | 0.4373*** | 0.3661*** | 0.4146*** | 0.0738*** | 0.0639*** | 0.2396*** | 0.2105*** | 0.1917*** | -0.0583*** | 0.3458*** |
| ScF5 | -0.0405* | 0.4961*** | -0.1193*** | 0.1785*** | 0.0426** | 0.0845*** | 0.0555** | 0.4947*** | 0.0466** | 0.1052*** | -0.0234 | -0.0247 | -0.0287 | 0.0346 | 0.1284*** |
| Note: *p < 0.1, **p < 0.05, ***p < 0.01  Total score: Peds QL 4.0 Total score; PF: Physical Functioning; EF: Emotional Functioning; SF: Social Functioning; ScF: School Functioning.  PF1: Hard to walk more than one block,PF2: Hard to run,PF3: Hard to do sports activity or exercise,PF4: Hard to lift something heavy,PF5: Hard to take a bath or shower,PF6: Hard to do chores around the house,PF7: Hurt or ache,PF8: Low energy,EF1: Feel afraid or scared,EF2: Feel sad or blue,EF3: Feel angry,EF4: Trouble sleeping,EF5: Worry about what will happen,SF1: Trouble getting along with others,SF2: Others not wanting to be friends,SF3: Teased,SF4: Not able to do things that others can do,SF5: Hard to keep up when play with others,ScF1: Hard to pay attention in class,ScF2: Forget things,ScF3: Trouble keeping up with schoolwork,ScF4: Miss school because of not feeling well,ScF5: Miss school to go to the doctor or hospital. | | | | | | | | | | | | | | | |

Appendix 1b The Spearman's correlation coefficient between variables

| Variables | PF8 | EF1 | EF2 | EF3 | EF4 | EF5 | SF1 | SF2 | SF3 | SF4 | SF5 | ScF1 | ScF2 | ScF3 | ScF4 | ScF5 |
| --- | --- | --- | --- | --- | --- | --- | --- | --- | --- | --- | --- | --- | --- | --- | --- | --- |
| PF8 | 1 |  |  |  |  |  |  |  |  |  |  |  |  |  |  |  |
| EF1 | 0.0906*** | 1 |  |  |  |  |  |  |  |  |  |  |  |  |  |  |
| EF2 | 0.0654*** | 0.7753*** | 1 |  |  |  |  |  |  |  |  |  |  |  |  |  |
| EF3 | 0.0607*** | 0.7571*** | 0.9578*** | 1 |  |  |  |  |  |  |  |  |  |  |  |  |
| EF4 | 0.4899*** | -0.0452** | -0.1201*** | -0.0966*** | 1 |  |  |  |  |  |  |  |  |  |  |  |
| EF5 | 0.0960*** | 0.1766*** | 0.1150*** | 0.1082*** | 0.0347 | 1 |  |  |  |  |  |  |  |  |  |  |
| SF1 | 0.0909*** | 0.4458*** | 0.3509*** | 0.3476*** | 0.0522** | 0.2753*** | 1 |  |  |  |  |  |  |  |  |  |
| SF2 | 0.2412*** | 0.3751*** | 0.3094*** | 0.3105*** | 0.2294*** | 0.3798*** | 0.6891*** | 1 |  |  |  |  |  |  |  |  |
| SF3 | 0.2875*** | 0.3936*** | 0.3371*** | 0.3395*** | 0.2671*** | 0.3725*** | 0.7132*** | 0.907*** | 1 |  |  |  |  |  |  |  |
| SF4 | 0.0084 | 0.2546*** | 0.1957*** | 0.1786*** | 0.1188*** | 0.1884*** | 0.1516*** | 0.0146 | 0.0027 | 1 |  |  |  |  |  |  |
| SF5 | -0.0077 | 0.2051*** | 0.1295*** | 0.1267*** | 0.0087 | 0.2429*** | 0.1346*** | 0.0425** | 0.0396* | 0.7065*** | 1 |  |  |  |  |  |
| ScF1 | 0.1077*** | 0.3367*** | 0.3269*** | 0.3331*** | 0.0587*** | 0.1408*** | 0.5988*** | 0.538*** | 0.5910*** | 0.0637*** | 0.0434** | 1 |  |  |  |  |
| ScF2 | 0.0708*** | 0.2173*** | 0.1662*** | 0.1690*** | 0.0058 | 0.3100*** | 0.4402*** | 0.5688*** | 0.5101*** | 0.1443*** | 0.1358*** | 0.3589*** | 1 |  |  |  |
| ScF3 | 0.0129 | 0.2176*** | 0.2171*** | 0.2172*** | 0.1288*** | 0.2393*** | 0.1722*** | 0.1709*** | 0.1808*** | 0.3551*** | 0.3108*** | 0.1120*** | 0.1564*** | 1 |  |  |
| ScF4 | 0.5597*** | 0.2579*** | 0.2436*** | 0.2607*** | 0.6123*** | 0.1207*** | 0.2059*** | 0.3878*** | 0.4384*** | 0.1121*** | 0.0307 | 0.2048*** | 0.1157*** | 0.1671*** | 1 |  |
| ScF5 | 0.1731*** | 0.1265*** | 0.1082*** | 0.1084*** | 0.1647*** | -0.0613*** | 0.0256 | 0.0509** | 0.0600*** | 0.0421* | 0.0406* | 0.0558*** | -0.0228 | 0.0563*** | 0.3141*** | 1 |
| Note: *p < 0.1, **p < 0.05, ***p < 0.01  Total score: Peds QL 4.0 Total score; PF: Physical Functioning; EF: Emotional Functioning; SF: Social Functioning; ScF: School Functioning.  PF1: Hard to walk more than one block,PF2: Hard to run,PF3: Hard to do sports activity or exercise,PF4: Hard to lift something heavy,PF5: Hard to take a bath or shower,PF6: Hard to do chores around the house,PF7: Hurt or ache,PF8: Low energy,EF1: Feel afraid or scared,EF2: Feel sad or blue,EF3: Feel angry,EF4: Trouble sleeping,EF5: Worry about what will happen,SF1: Trouble getting along with others,SF2: Others not wanting to be friends,SF3: Teased,SF4: Not able to do things that others can do,SF5: Hard to keep up when play with others,ScF1: Hard to pay attention in class,ScF2: Forget things,ScF3: Trouble keeping up with schoolwork,ScF4: Miss school because of not feeling well,ScF5: Miss school to go to the doctor or hospital. | | | | | | | | | | | | | | | | |
